# Efficacy of Goshajinkigan for Peripheral Neurotoxicity of Oxaliplatin in Patients with Advanced or Recurrent Colorectal Cancer

**DOI:** 10.1093/ecam/nep200

**Published:** 2011-01-11

**Authors:** Toru Kono, Noriaki Mamiya, Naoyuki Chisato, Yosiaki Ebisawa, Hirotaka Yamazaki, Jiro Watari, Yasuhiro Yamamoto, Shigetaka Suzuki, Toshiyuki Asama, Kazunori Kamiya

**Affiliations:** ^1^Division of Gastroenterologic and General Surgery, Department of Surgery, Asahikawa Medical University, 2-1 Midorigaoka-higashi, Asahikawa, Hokkaido 078-8510, Japan; ^2^Division of Cancer Chemotherapy Center, Higashiasahikawa Hospital, Asahikawa, Japan; ^3^Department of Surgery, Kushiro City Medical Association Hospital, Kushiro, Japan; ^4^Deptartment of Gastroenterology, Kushiro City Medical Association Hospital, Kushiro, Japan; ^5^Department of Surgery, Kobayashi Hospital, Kitami, Japan; ^6^Department of Surgery, Karasawa Hospital, Asahikawa, Japan

## Abstract

Peripheral neurotoxicity is the major limiting factor for oxaliplatin therapy. Goshajinkigan (GJG), a traditional Japanese herbal medicine, was recently shown to be effective in protecting against the neurotoxicity of taxanes in Japan. We retrospectively investigated the effect of GJG on peripheral neurotoxicity associated with oxaliplatin therapy. Ninety patients with metastatic colorectal cancer that received FOLFOX4 or modified FOLFOX6 therapy were assigned to receive one of the following adjuncts: oral GJG at 7.5 g day^−1^ (Group A, *n* = 11), intravenous supplementation of calcium gluconate and magnesium sulfate (1 g each before and after FOLFOX) (Group B, *n* = 14), combined GJG and calcium gluconate and magnesium sulfate therapies (Group C, *n* = 21), or no concomitant therapy (Group D, *n* = 44). The incidence of peripheral neurotoxicity was investigated when the cumulative dose of oxaliplatin exceeded 500 mg m^−2^. When the cumulative dose of oxaliplatin exceeded 500 mg m^−2^, the incidence of neuropathy (all grades) in Groups A–D was 50.0%, 100%, 78.9%, and 91.7%, respectively. It was lowest in the group that received GJG alone. Concomitant administration of GJG reduced the neurotoxicity of oxaliplatin in patients that received chemotherapy for colorectal cancer.

## 1. Introduction

In recent years, the standard chemotherapy for advanced/recurrent colorectal cancer is a continuous intravenous infusion of 5-fluorouracil (5-FU) combined with either oxaliplatin (FOLFOX, FOLFOX4 or modified FOLFOX6) or irinotecan (FOLFIRI) [1–3]. Acute and persistent peripheral neuropathy is the characteristic of oxaliplatin therapy [4], and the oxaliplatin dose must be limited to avoid toxicity. The prevalence of peripheral neurotoxicity increases with the total accumulated dose of oxaliplatin, and often interferes with the continuation of FOLFOX therapy [5]. Gamelin et al. [6, 7] reported that administration of calcium gluconate and magnesium sulfate (Ca/Mg) before and after oxaliplatin therapy could alleviate peripheral neurotoxicity. Other similar treatments have been described, including carbamazepine [8–10] or glutathione [11], but an effective remedy for peripheral neurotoxicity related to oxaliplatin therapy has not yet been established.

Goshajinkigan (GJG) is an extracted traditional Japanese herbal medicine (Kampo) that is mainly used for the improvement of symptoms like numbness, cold sensation and limb pain associated with diabetic neuropathy [12–15]. Moreover, Mamiya et al. [16] and Shindo et al. [17] recently reported that peripheral neurotoxicity due to oxaliplatin was relieved by administration of GJG in patients with advanced colorectal cancer that were receiving FOLFOX therapy.

We conducted the present retrospective study to compare the efficacy of GJG with that of Ca/Mg for alleviation of peripheral neurotoxicity in patients with advanced or recurrent colorectal cancer that received either FOLFOX4 therapy or modified FOLFOX6 (mFOLFOX6) therapy at our hospital and affiliated institutions in Japan.

## 2. Patients and Methods

### 2.1. Patients

This retrospective analysis included 90 patients with advanced or recurrent colorectal cancer that had received either FOLFOX4 or mFOLFOX6 therapy from August 2005 to January 2008 at our hospital and five affiliated institutions. Patients were classified into the following four groups: chemotherapy + GJG, chemotherapy + Ca/Mg, chemotherapy + GJG + Ca/Mg and chemotherapy alone. Full ethical approval for this study has been obtained from all of each responsible Ethics Committees in each hospital according to Japanese Ministry of Health, Labour and Welfare guidelines. All patients provided written informed consent. All records will be kept confidential and the patient's; name will not be released at any time.

### 2.2. Chemotherapy

On Day 1, patients treated with FOLFOX4 received a 2-h intravenous infusion of oxaliplatin (85 mg m^−2^) combined with levofolinate (1-LV, 100 mg m^−2^), followed by a bolus injection of 5-FU (400 mg m^−2^), and then continuous infusion of 5-FU (600 mg m^−2^) for 22 h. On Day 2, 1-LV (100 mg m^−2^) was administered in a 2-h intravenous infusion, and then a bolus of 5-FU was administered (400 mg m^−2^), followed by a 22-h continuous 5-FU infusion (600 mg m^−2^). This regimen comprised one course of therapy and was repeated once every 2 weeks.

On Day 1, patients treated with mFOLFOX6 therapy received a 2-h intravenous infusion of oxaliplatin (85 mg m^−2^) combined with 1-LV (100 mg m^−2^), followed by a rapid intravenous infusion of 5-FU (400 mg m^−2^), and then a 46-h continuous infusion of 5-FU (2400 mg m^−2^). This regimen comprised one course of therapy and was repeated once every 2 weeks.

GJG (7.5 mg day^−1^ divided into 2-3 doses) was administered during FOLFOX therapy, given orally before meals or between meals on a daily basis. Ca and Mg (1 g each) were administered before and after FOLFOX therapy by intravenous infusion.

### 2.3. Endpoints and Evaluation

Each group was evaluated to determine the total dose of oxaliplatin, the median and mean numbers of courses, the incidence of each grade of peripheral neuropathy, the incidence of peripheral neuropathy when the total dose of oxaliplatin exceeded 500 mg m^−2^, the total dose of oxaliplatin at which 50% of patients showed peripheral neuropathy and the time to treatment failure (TTF). Peripheral neuropathy evaluations were based on the Neurotoxicity Criteria of DEBIOPHARM (DEB-NTC) [18]. The assessment of the occurrence of peripheral neuropathy in relation to the total dose of oxaliplatin, and the TTF comparisons were based on Kaplan-Meier analyses. The attending physicians assessed the anti-tumor effect of chemotherapy with the new Guidelines for Evaluation of the Response to Treatment in Solid Tumors (RECIST) [19]. In this retrospective study, Groups A–C were compared with Group D (no adjunct treatment) with the log-rank test to identify differences in the incidence of peripheral neuropathy and TTF. Differences observed among groups in the incidence of peripheral neuropathy at a total oxaliplatin dose >500 mg m^−2^ and differences in the anti-tumor activity were assessed with the chi-square test.

## 3. Results

### 3.1. Patient Characteristics

There were an unequal number of patients in each group (Table 1). Group D (no concomitant therapy) was the largest group and Group A (GJG alone) was the smallest group. There were no between-group differences of sex (*P*-values are for Groups A (*P* = .890), B (*P* = .223) and C (*P* = .745) versus Group D by the *χ* ^2^-test) and age (*P*-values are for Groups A (*P* = .954), B (*P* = .470), and C (*P* = .790) versus Group D by the *t-*test). The performance status (PS) was evaluated with The Eastern Cooperative Oncology Group criteria. A higher percentage of Group A patients had a PS = 1 compared with the other groups. None of the patients in Groups A or B had a PS = 2, but there were no significant differences in PS between the groups (*P*-values are for Groups A (*P* = .373), B (*P* = .316) and C (*P* = .702) versus Group D by the *χ* ^2^-test). There were no differences in the locations of the primary and metastatic tumors (*P*-values are for Groups A (*P* = .498), B (*P* = .431) and C (*P* = .993) versus Group D by the *χ* ^2^-test). 

### 3.2. FOLFOX Therapy

The chemotherapy regimen most commonly administered in Group A was mFOLFOX6, and all of the patients in Groups B and C received mFOLFOX6 therapy. Only Group D had a relatively large number of patients that received FOLFOX4 therapy.  Table 2 shows the median and mean total doses of oxaliplatin for each group. Both the median and mean doses were highest in Group A, followed by Groups C, D and B in descending order. Compared with Group D, Group B had a smaller percentage of patients (*P* = .004 by the *χ* ^2^-test.) that received a total dose of oxaliplatin exceeding 500 mg m^−2^, but there was no difference between the other two groups (*P* = .466 and .366 by the *χ* ^2^-test.). Groups A, C and D had similar medians and mean numbers of courses, but Group B had the lowest number of courses (Table 2).

### 3.3. Peripheral Neuropathy

**Figure 1:**
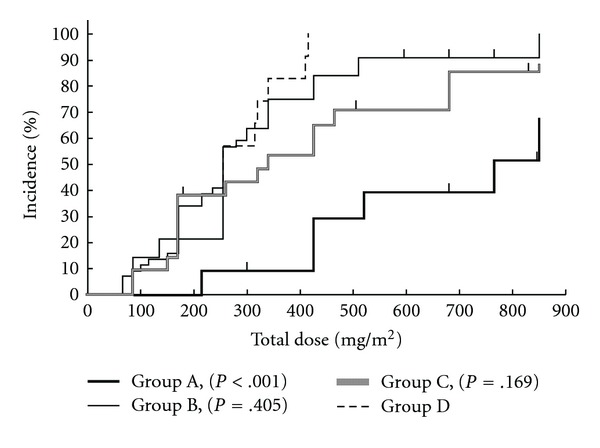
Kaplan-Meier analysis of Grade 1 or worse peripheral neuropathy in relation to the total dose of oxaliplatin. Group A, GJG; Group B, calcium gluconate (Ca) and magnesium sulfate (Mg); Group C, GJG + Ca/Mg; Group D, no therapy. *P*-values are for comparison to Group D with the log-rank test.

**Figure 2:**
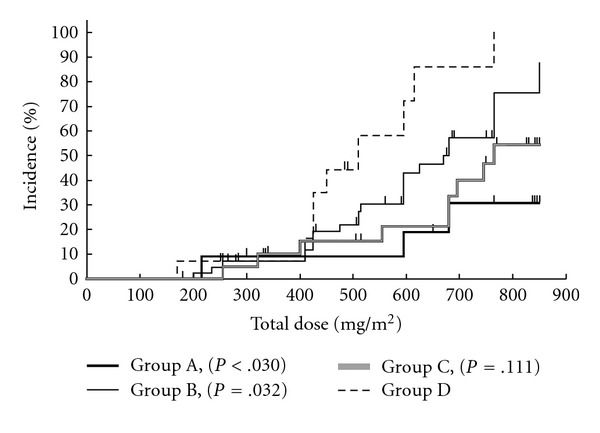
Kaplan-Meier analysis of Grade 2 or worse peripheral neuropathy in relation to the total dose of oxaliplatin. Group A, GJG; Group B, calcium gluconate (Ca) and magnesium sulfate (Mg); Group C, GJG + Ca/Mg; Group D, no therapy. *P*-values are for comparison to Group D with the log-rank test.

**Figure 3:**
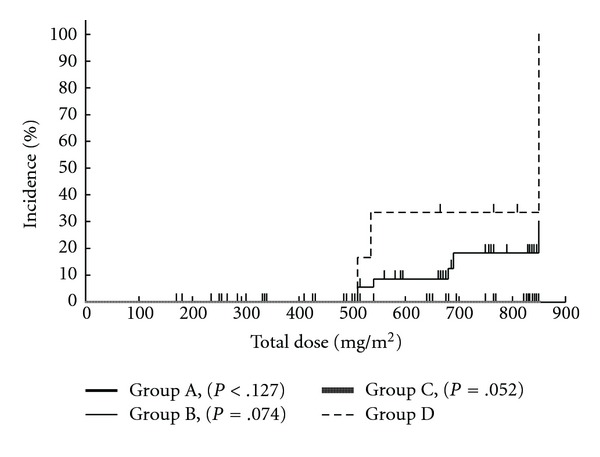
Kaplan-Meier analysis of Grade 3 peripheral neuropathy in relation to the total dose of oxaliplatin. Group A, GJG; Group B, calcium gluconate (Ca) and magnesium sulfate (Mg); Group C, GJG + Ca/Mg; Group D, no therapy. *P*-values are for comparison to Group D with the log-rank test.

Kaplan-Meier analyses showed that peripheral neuropathy of Grade 1 or worse (Figure 1) and Grade 2 or worse (Figure 2) occurred less frequently in Group A compared with the other groups; the difference was most marked for neuropathy of Grade 1 or worse. Peripheral neuropathy of Grade 3 (Figure 3) did not occur in either Groups A or C, the two groups that received GJG therapy.

The incidence of peripheral neuropathy at a total oxaliplatin dose >500 mg m^−2^ was lower in the two groups given GJG (Group A and Group C; Table 3). In Group A, there were no cases of Grades 2 or 3 peripheral neuropathy. In Group B (Ca/Mg alone), the overall incidence of neuropathy was comparable to that in Group D (no concomitant therapy), but Group B had a higher rate of Grade 3 peripheral neuropathy (Table 3).

The total dose of oxaliplatin at which 50% of the patients developed peripheral neuropathy was 765 mg m^−2^ in Group A for Grade 1 or worse neuropathy (Table 4). In Group A, 50% level was not reached for Grade 2 or worse neuropathy; this suggested that a higher oxaliplatin dose could be administered to Group A when compared to the other groups before peripheral neurotoxicity occurred.

### 3.4. Treatment Failure and Discontinuation

**Figure 4:**
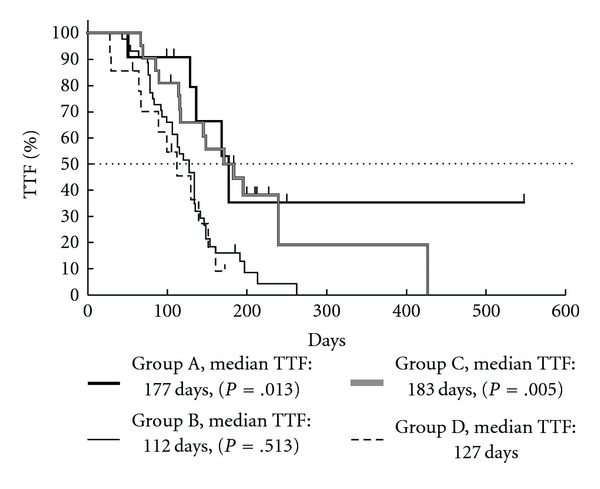
Kaplan-Meier analysis of TTF. Group A, GJG; Group B, calcium gluconate (Ca) and magnesium sulfate (Mg); Group C, GJG + Ca/Mg; Group D, no therapy. *P*-values are for comparison to Group D with the log-rank test.

The TTF was ∼2 months longer in the groups given GJG than in the groups without GJG (Figure 4). The rate of discontinuation of treatment due to tumor progression was 18.2% and 20.5% in patients that did not receive Ca/Mg (Groups A and D, resp.), but only 7.1% and 4.8% in those that received Ca/Mg (Groups B and C, resp.) (Table 5). In Group A, no patients discontinued therapy due to peripheral neurotoxicity; in the other groups, the rates ranged from 19.0% to 42.9%. However, Group A showed a higher discontinuation due to hematological toxicity than the other groups. There were no discontinuations due to patient refusal or change of therapy in the groups administered GJG. Also, in Group A, nearly half the patients continued the treatment throughout the study (Table 5).

### 3.5. Tumor Response

The response rate was 54.5% (6/11) in Group A, 35.7% (5/14) in Group B, 42.9% (9/21) in Group C and 45.5% (20/44) in Group D. The disease control rate (stable disease or better) was 90.9% (10/11) in Group A, 71.4% (10/14) in Group B, 90.5% (19/21) in Group C and 88.6% (39/44) in Group D (Table 6).

## 4. Discussion

Peripheral neurotoxicity is a characteristic adverse effect of oxaliplatin [20]. It is a major obstacle to continuing treatment with regimens that contain this agent; for example, FOLFOX. The main symptoms of peripheral neurotoxicity are typically paresthesia and dysestheia of the extremities induced by exposure to cold. These symptoms occur in 85%–95% of patients, and the symptom duration becomes longer with increasing repetitions of chemotherapy courses. It has been shown that an increase in the total dose of oxaliplatin leads to the occurrence of pain and sensory dysfunction. De Gramont et al. [5] reported that functional impairment occurred in 10% of patients at a total oxaliplatin dose of 850 mg m^−2^, and this increased to 20% at a total dose of 1020 mg m^−2^. Thus, it is important to control peripheral neurotoxicity in order to allow continued administration of oxaliplatin. However, control of this side effect is difficult, because the mechanisms underlying the development of neuropathy have not been clarified.

Gamelin et al. [6] suggested that a possible mechanism may be the effect of oxalate, a metabolite of oxaliplatin, on neuronal sodium channels. However, based on this hypothesis, treatment with a sodium channel blocker did not achieve satisfactory results [20]. This suggested that sodium channels may only be involved in acute peripheral neurotoxicity. Neuronal damage has also been attributed to the accumulation of platinum in the dorsal root ganglion based on the results from animal experiments [20]. Thus multiple mechanisms may be involved.

**Figure 5:**
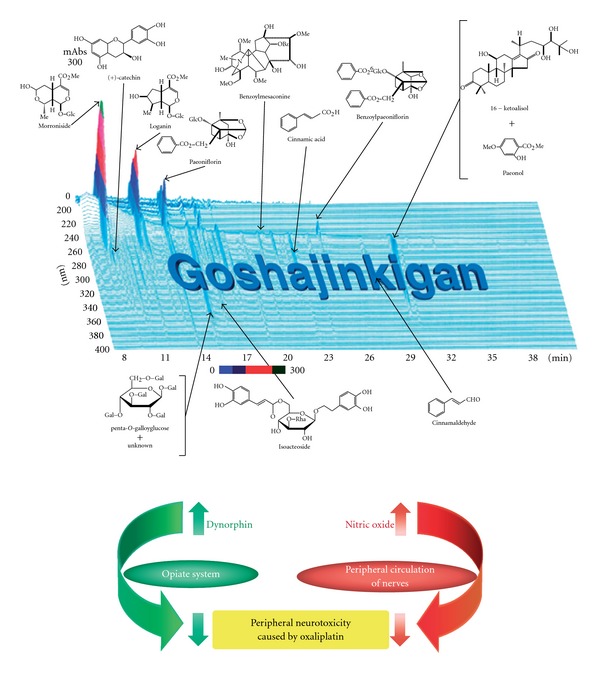
Two mechanisms of pharmacological actions of GJG for peripheral neurotoxicity. This shows a representative 3D high performance liquid chromatography of the GJG formulation. Two mechanisms are suggested by which GJG may alleviate peripheral neurotoxicity caused by oxaliplatin.

GJG is comprised of 10 herbs and each contains numerous active ingredients (Figure 5). Thus, in Western medical terms, GJG is a complex drug, and its overall pharmacological action is difficult to explain. Until recently, many Japanese people harbored prejudice toward Kampo medicine, doubted their efficacy and showed little interest in their mechanisms [21]. This ignorance of the potential benefits of some herbal medicines is hardly a rare phenomenon, because skeptics of herbal medicine abound wherever herbal medications are used, although herbal medicine has been used throughout the world since time immemorial [22]. Two mechanisms have been suggested by which GJG may alleviate peripheral neurotoxicity (Figure 5) [23–25]. The first is that GJG promotes the release of dynorphin, and thus improves numbness/pallesthesia via the opiate system. The second is that GJG promotes nitric oxide production, and thus improves the circulation and the blood supply to the nerves.

The present study was a retrospective analysis of peripheral neuropathy inhibition in patients that received FOLFOX therapy combined with GJG, Ca/Mg, GJG + Ca/MG or no concomitant drug. Although the number of patients was small, we found that the incidence of peripheral neuropathy was markedly lower in the groups that received GJG when compared with those that did not receive GJG; moreover, there was no Grade 3 peripheral neuropathy in the patients given GJG. In addition, more courses of chemotherapy could be given to patients that received GJG than to those not given GJG; thus, the former also received a higher total dose of oxaliplatin. Furthermore, the TTF was longer in patients that received GJG and there were fewer discontinuations due to peripheral neuropathy than for the other groups. In the group of patients given GJG alone (Group A), almost half the patients had continued oxaliplatin therapy throughout the study, and none discontinued treatment due to peripheral neurotoxicity. Group A showed a higher discontinuation due to hematological toxicity than the other groups. As far as we know, it has never been reported that hematological toxicity was observed in the patients who used GJG in Japan. GJG has been safely used for 25 years in Japan for the improvement of symptoms of numbness, cold sensation and pain of the extremities associated with diabetic neuropathy [12–15]. Therefore, hematological toxicity is not likely to have theadverse effect of GJG. The reason why Group A showed a higher discontinuation due to hematological toxicity is probably because Group A patients received higher dose of total oxaliplatin and 5-FU than other groups. In addition, that group had higher tumor response rates and disease control rates than the other groups, indicating that GJG treatment did not impair anti-tumor activity. Based on these results, we concluded that concomitant administration of GJG contributed to the inhibition of peripheral neurotoxicity and prolonged treatment with oxaliplatin.

Several authors have previously reported suppression of peripheral neurotoxicity by Ca/Mg; however, we could not confirm this effect in this study. In fact, the patients that received GJG + Ca/Mg developed worse neuropathy than those that received GJG alone. Accordingly, we suggest that GJG alone (rather than combined with Ca/Mg) may be more effective for suppression of peripheral neurotoxicity. In addition, the tumor response rate was lower in the group that received GJG + Ca/Mg than in the other groups; this suggested that some interaction may have occurred when GJG and Ca/Mg were combined.

A limitation of the present study was that it was a retrospective review. Also, the number of patients was small, and some of the baseline characteristics differed between the groups. Nevertheless, our findings suggested that the peripheral neurotoxicity of oxaliplatin could be suppressed by administration of GJG. It will be necessary to confirm the usefulness of GJG by performing larger prospective studies in the future [26].

## Figures and Tables

**Table 1:** Patient characteristics for the four groups.

	Group A (*n* = 11)	Group B (*n* = 14)	Group C (*n* = 21)	Group D (*n* = 44)	Total (*n* = 90)
Sex					
Male	7	6	12	27	52
Female	4	8	9	17	38
Age, median (range)	62 (47–78)	61.5 (54–75)	63 (36–82)	64 (43–87)	63 (36–87)
Body weight, median (range)	59 (41–76)	60 (40–75)	58 (38–77)	59 (39–76)	59 (38–77)
PS					
0	7	13	15	38	73
1	4	1	4	3	12
2	0	0	2	3	5
Primary tumor					
Colon	4	5	10	21	40
Rectum	7	9	11	23	50
Metastasis					
Liver	9	9	12	28	58
Lung	3	5	4	18	30
Lymph nodes	0	2	1	1	4
Other	2	4	7	8	21

Group A, GJG; Group B, Ca/Mg; Group C, GJG + Ca/Mg; Group D, no therapy.

GJG, goshajinkigan; Ca, calcium gluconate; Mg, magnesium sulfate; PS, performance status.

**Table 2:** Details of FOLFOX therapy.

	Group A (*n* = 11)	Group B (*n* = 14)	Group C (*n* = 21)	Group D (*n* = 44)	Total (*n* = 90)
FOLFOX					
FOLFOX4	4	0	0	33	37
mFOLFOX6	7	14	21	11	53
Cumulative oxaliplatin Dose (mg m^−2^)					
Median	807.5	500.0	750.0	680.0	680.0
Mean	726.3	534.3	686.7	625.0	632.3
Range	300–850	170–850	180–850	235–850	170–850
Total oxaliplatin dose ≥500 mg m^−2^	90.9%	42.9%	90.5%	81.8%	78.9%
Percentage of patients	(*n* = 10)	(*n* = 6)	(*n* = 19)	(*n* = 36)	(*n* = 71)
in group					
No. of courses					
Median	10.0	6.0	10.0	8.0	8.5
Mean	8.9	6.8	8.8	7.9	8.0
Range	4–10	2–10	3–10	3–10	2–10

Group A, GJG; Group B, Ca/Mg; Group C, GJG + Ca/Mg; Group D, no therapy.

GJG, goshajinkigan; Ca, calcium gluconate; Mg, magnesium sulfate.

**Table 3:** Frequency of peripheral neuropathy at a total oxaliplatin dose of 500 mg m^−2^.

	Group A,		Group B,		Group C,		Group D (*n* = 36)
	(*n* = 10)		(*n* = 6)		(*n* = 19)		
	Percentage	*P*-value	Percentage	*P*-value	Percentage	*P*-value	
All grades	50.0	.002	100	.463	78.9	.178	91.7
Grade 2	0	.130	16.7	.873	5.3	.156	19.4
Grade 3	0	.345	33.3	.080	0	.196	8.3

Group A, GJG; Group B, Ca/Mg; Group C, GJG + Ca/Mg; Group D, no therapy.

*P*-values are for Groups A, B and C versus. Group D by the *χ* ^2^-test.

GJG, goshajinkigan; Ca, calcium gluconate; Mg, magnesium sulfate.

*n*: numbers are patients received over 500 mg m^−2^ dose of total oxaliplatin.

**Table 4:** Total dose of oxaliplatin at which 50% of patients developed neuropathy.

	Group A	Group B	Group C	Group D
	(*n* = 11)	(*n* = 14)	(*n* = 21)	(*n* = 44)
Grade ≥1	765	255	340	255
Grade ≥2	Not reached	510	765	670
Grade 3	—	850	—	Not reached

Total oxaliplatin doses are shown in mg/m^2^.

Grade ≥1, Grade 1 or worse neuropathy; Grade ≥2, Grade 2 or worse neuropathy;

Group A, GJG; Group B, Ca/Mg; Group C, GJG + Ca/Mg; Group D, no therapy.

GJG, goshajinkigan; Ca, calcium gluconate; Mg, magnesium sulfate.

**Table 5:** Reasons for discontinuation of therapy.

	Group A,	Group B,	Group C,	Group D,	Total,
	Pts (%)	Pts (%)	Pts (%)	Pts (%)	Pts (%)
Progressive disease	2 (18.2)	1 (7.1)	1 (4.8)	9 (20.5)	13 (14.4)
Others					
Neuropathy	0	6 (42.9)	4 (19.0)	10 (22.7)	20 (22.2)
Myelosuppression	2 (18.2)	1 (7.1)	0	3 (6.8)	6 (6.7)
Allergy	0	0	2 (9.5)	3 (6.8)	5 (5.6)
Other toxicities	1 (9.1)	2 (14.3)	7 (33.3)	9 (20.5)	19 (21.1)
Resection	1 (9.1)	2 (14.3)	3 (14.3)	1 (2.3)	7 (7.8)
Patient refusal	0	1 (7.1)	0	0	1 (1.1)
Change of therapy	0	0	0	5 (11.4)	5 (5.6)
Continuing	5 (45.5)	1 (7.1)	4 (19.0)	4 (9.1)	14 (15.6)

Group A, GJG; Group B, Ca/Mg; Group C, GJG + Ca/Mg; Group D, no therapy.

GJG, goshajinkigan; Ca, calcium gluconate; Mg, magnesium sulfate.

**Table 6:** Tumor response to treatment (RECIST).

	Group A	Group B	Group C	Group D
CR	0	0	0	0
PR	6	5	9	20
SD	4	5	10	19
PD	1	3	1	3
NE	0	1	1	2
Response Rate	6 (54.5%)	5 (35.7%)	9 (42.9%)	20 (45.5%)
(CR+PR)	(*P* = .589)	(*P* = .522)	(*P* = .844)	
Disease Control Rate	10 (90.9%)	10 (71.4%)	19 (90.5%)	39 (88.6%)
(CR+PR+PD)	(*P* = .829)	(*P* = .121)	(*P* = .823)	

Group A: GJG, Group B: Ca/Mg, Group C: GJG+Ca/Mg, Group D: no therapy.

CR: complete response; PR: partial response; SD: stable disease; PD: progressive disease; NE: no evaluate.

*P*-values are for Groups A, B, and C versus. Group D by the *χ* ^2^-test.

GJG: goshajinkigan, Ca: calcium gluconate, Mg: magnesium sulfate.
